# Evaluation of an automated dish preparation system for IVF and embryo culture using a mouse mode

**DOI:** 10.1038/s41598-023-43665-y

**Published:** 2023-10-01

**Authors:** Yan Zhu, Huai L. Feng, Man-Xi Jiang

**Affiliations:** 1 Medical Experiment Center, Guangdong Second Provincial General Hospital, Guangzhou, People’s Republic of China; 2 New York Fertility Center, New York-Presbyterian Healthcare System Affiliate Weill Cornell Medical College, New York, USA; 3 Center for Reproductive Medicine, Guangdong Second Provincial General Hospital, Guangzhou, People’s Republic of China

**Keywords:** Biological techniques, Biotechnology, Developmental biology, Health occupations

## Abstract

Manual dish preparation for IVF in human fertility clinics or animal laboratories heavily relies on embryologists' experience, which can lead to occupational illness due to long-term and monotonous operation. Therefore, introducing an automated technique to replace traditional methods is crucial for improving working efficiency and reducing work burden for embryologists. In the current study in the mouse, both manual and automated methods were used to prepare IVF or embryo culture dishes. A one-way analysis of variance was conducted to compare several factors, including preparation time, qualified rates, media osmolality of dishes, fertilization rates, and embryonic development to assess the efficiency and potential of automated preparation. The results showed that automation system significantly reduced the required time and increased the efficiencies and qualified rates of dish preparation, especially for embryo culture dishes, without significantly altering medium osmolalities. There were no significant differences between two preparations in fertilization rates and embryo development in mice. Thus, automated dish preparation can improve working efficiency and qualified rates while maintaining fertilization rates and subsequent embryonic development without compromising osmolality stability of medium. It presents a superior alternative to manual preparation, reducing the workload of embryologists and facilitating the standardization of operational procedures.

## Introduction

Manual preparation of IVF and embryo culture dishes is a routine in IVF or reproductive biology laboratory^1,2^, as for manual preparation in large human IVF centres with more cycles (e.g. more than thousands of cycles per year), dozens of or even hundreds of culture dishes need to be daily prepared. Without doubt which is a huge workload for embryologists, and the long-term and repetitive nature of this work can easily produce huge physical and mental pressure, which can be damaging to embryologists' health. If an automated method is introduced into IVF laboratory, the work efficiency may be improved without affecting the research or clinical outcomes. To some extent, to partially replace manual task by automation can also reduce workloads for embryologists, is helpful to maintain their physical or mental health, and save much time to perform the other important tasks.

During gamete or embryo culture, the fluctuation of pH value and osmolality of media can impact oocyte quality^3^ and embryo development^4^, culture dishes kept out of the incubator for even brief periods can result in the pH rising above 7.4 in media^5,6^, but the formulated media are able to resist these changes of pH through the buffering of CO_2_ at certain concentration in CO_2_ incubator. However, osmolality as another important influencing factor during embryo culture^7,8^, its stability may inadvertently be affected by lab operations such as dish preparation, microdroplet volume^9,10^, use of mineral oil^11,12^ and design of the dish^13,14^, and particularly the osmolality rise is an irreversible adverse stress for embryos cultivated in microdroplets.

Therefore, an automated dish preparation needs to be invented to provide a more standard and repeatable process that can help embryologists achieve consistent outcomes with a higher work efficiency, which can be used to reduce the workloads, improve the precision of medium droplets including size and location, decrease the influence of personnel factors on osmolality of culture media. Moreover, personnel and equipment-related factors, such as manual operation or the repeated use of pipette tips, can increase the risk of microbial contamination in manual dish preparation^15^. However, a completely enclosed automated preparation system can eliminate this potential contamination risks associated with manual pipetting.

In our present program an automated dish preparation system was developed, and its working mechanism and operation process were introduced in detail. In this study, dishes for IVF and embryo culture were prepared by manual or automation, and time-consumed for individual dish, size uniformity and layout regularity of microdroplets, qualified rates of dish preparation, osmolality changes of culture media, fertilization and embryonic development in mice were evaluated in order to verify the efficiency and feasibility of this automation system.

## Results

### Difference of the required time between automated and manual preparation

**Figure 1:**
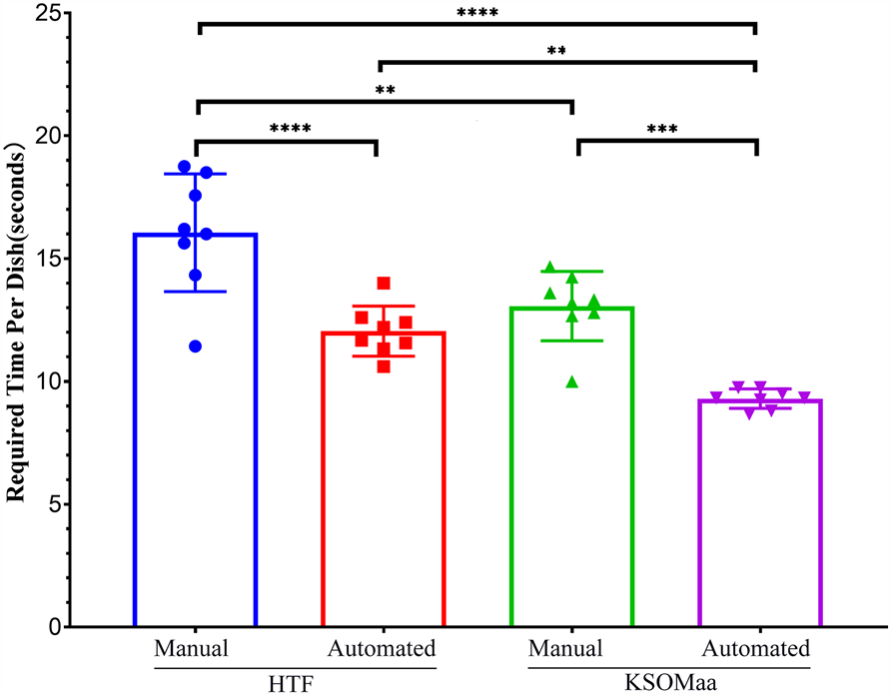
Comparison of the required time for preparing IVF and culture dish. The average time each dish of automatically prepared IVF dish (HTF) was 12.05 ± 1.02 s while that in manually prepared dish was 16.05 ± 2.39 s; there was a significant difference between two groups (*P* < 0.01). The average time each embryo of automatically prepared embryo culture dish (KSOMaa) was 9.30 ± 0.40 s, while that in manually prepared dish was 13.06 ± 0.40 s; there was a significant difference between two groups (*P* < 0.001). Additionally, the manual method required longer preparation time for both IVF and embryo culture dishes compared to the automated method, but no significant difference was observed between the preparation time of automatically prepared embryo culture dish and manually prepared IVF dish (*P* > 0.05). NS means no significant difference; “**” and “***” on the bars signify the p values are less than 0.01 and 0.001 respectively.

The average time per IVF dish in automated preparation group was 12.05 ± 1.02 s (n = 8) while that in manual preparation group was 16.05 ± 2.39 s (n = 8); there was a significant difference between two groups (*P* < 0.001, Fig. 1 and Supplemental Table S1). The results demonstrated that automated preparation of each IVF dish required significantly less time than manual preparation. The average time per embryo culture dish in automated preparation group was 9.30 ± 0.40 s (n = 8), while that in manual preparation group was 13.06 ± 1.41 s (n = 8); there was a significant difference between two groups (*P* < 0.0001, Fig. 1 and Supplemental Table S1), which suggested that automated preparation of each embryo culture dish also required markedly less time than manual preparation.

It suggested that this automated system could save more time for preparing IVF or culture dishes compared to the manual preparation.

### Droplet regularities and qualified rates of manually and automatedly prepared dishes

**Figure 2:**
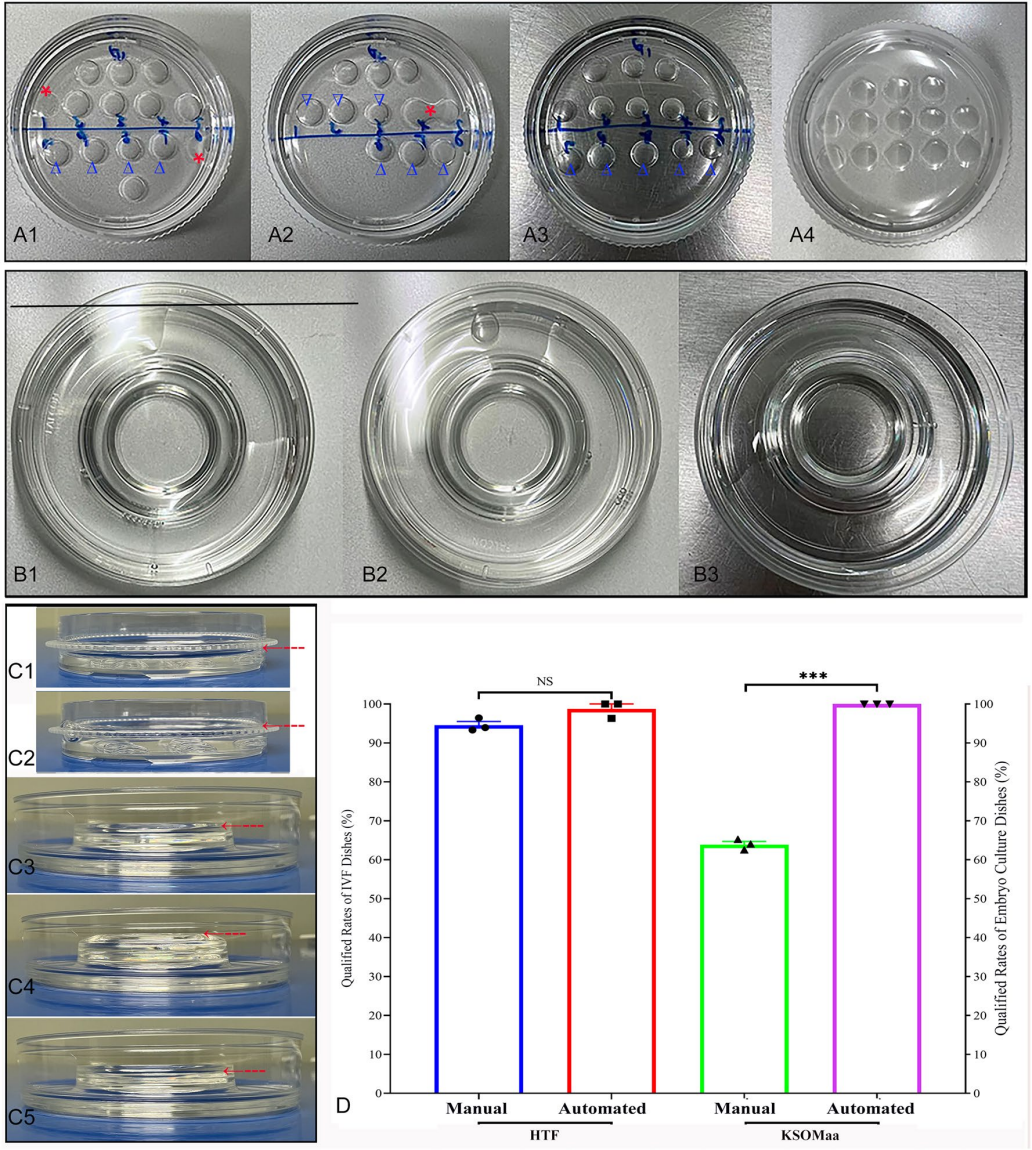
Representative photographs and qualified rates of dishes prepared by manual and automated procedures. In manual group, the sizes of microdroplets were different (Blue triangles; **A1**–**A3**), the arrangement of microdroplet was sometime irregular (**A2**–**A3**), the adjacent droplets easily merged (Red asterisk; **A2**), some droplets easily flatted to the edge of dishes (Red asterisk; **A1**), and the height (volume) of oil was occasionally too high (Red dashed line with arrow; **C2**). The sizes of and distances between different microdroplets were very consistent in automatically prepared dishes (**A4**). The qualified rates between manually and automatically prepared embryo culture dishes were up to 63.91 ± 1.36% and 100.00 ± 0.00%, respectively. These results indicate a significant difference between the two methods (*P* < 0.001). Overall, there were no significant differences in IVF dishes prepared by manual or automation (**B1**–**B3**), except for a few variations in the height of the medium (oil) layer (Red dashed line with arrow; **C4**–**C5**) in some dishes prepared by manual dispensing. There was no significant statistical difference in the qualified rates of IVF dishes between the manual and automated methods (94.57 ± 1.64% *vs* 98.77 ± 2.14%; *P* > 0.05). The red dashed line with arrow indicates the height of oil layer; NS means no significant difference; “**”, “***” and “****” on the bars signify the p values are less than 0.01, 0.001 and 0.0001 respectively.

During manual preparation of embryo culture dishes, the sizes of microdroplets were not always consistent (Fig. 2A1–A3), the arrangement of microdroplet was sometimes irregular (Fig. 2A2,A3), the adjacent droplets occasionally merged together (Fig. 2A2), some droplets easily flatted to the edge of dishes (Fig. 2A1) and the height (volume) of oil was occasionally high (Fig. 2C1,C2) or low. However, comparing with manual preparation, the sizes of and distances between different microdroplets were very consistent in automatically prepared embryo culture dishes (Fig. 2A4). The qualified rates between manually and automatically prepared embryo culture dishes were up to 63.91 ± 1.36% and 100.00 ± 0.00% (n = 3), respectively; and there was significant difference (Fig. 2D and Supplemental Table S2; *P* < 0.001).

In the IVF dishes prepared manually (Fig. 2B1,B2) and through automation (Fig. 2B3), there were no noticeable differences in the heights of the medium and oil layers, except for a few manually prepared dishes with excessively high or low oil layers (Fig. 2C3–C5). Additionally, there was no significant statistical difference (94.57 ± 1.64% *vs* 98.77 ± 2.14%; n = 3) in the qualified rates of IVF dishes between the manual and automated methods (Fig. 2D and Supplemental Table S2; *P* > 0.05).

### Osmolality differences of HTF in IVF dish and KSOMaa in embryo culture dish between manual and automated preparations

**Figure 3:**
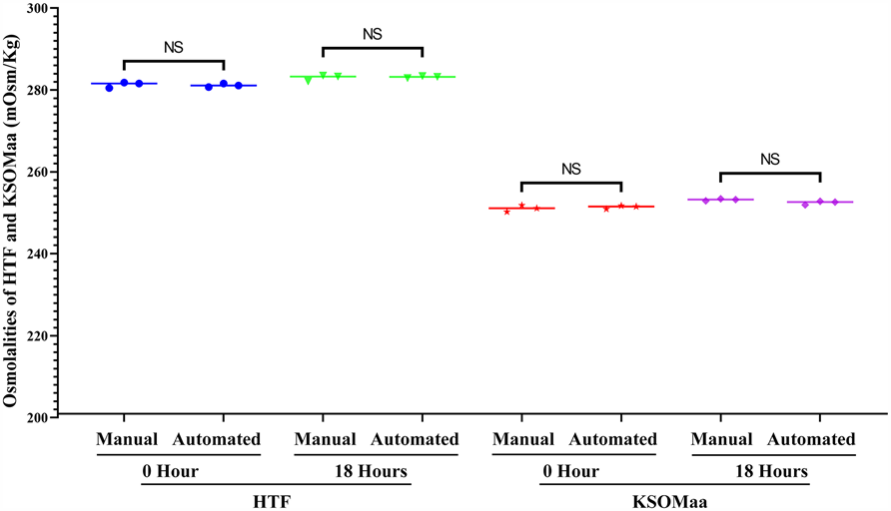
Osmolality changes in IVF and embryo culture dishes between manual and automated preparations. The initial osmolality between manual and automated group were 281.30 ± 0.70 *vs* 281.13 ± 0.45 mOsm/kg for HTF and 251.03 ± 0.80 *vs* 251.37 ± 0.42 mOsm/kg for KSOMaa; the osmolalites after overnight prewarming between manual and automated group were 282.97 ± 0.76 *vs* 283.17 ± 0.25 mOsm/kg for HTF and 253.17 ± 0.25 *vs* 252.43 ± 0.47 mOsm/kg for KSOMaa. No significant differences in osmolality whether in HTF or KSOMaa media were detected between manual and automated groups. NS means no significant difference.

The initial osmolalities (mean ± SD) between manual and automated group were 281.30 ± 0.70 *vs* 281.13 ± 0.45 mOsm/kg for HTF medium (n = 3) in IVF dishes, and 251.03 ± 0.80 *vs* 251.37 ± 0.42 mOsm/kg for KSOMaa medium (n = 3) in embryo culture dishes; the osmolalities after prewarming overnight (18 h) between manual and automated group were 282.97 ± 0.76 *vs* 283.17 ± 0.25 mOsm/kg for HTF medium (n = 3) in IVF dishes, and 253.17 ± 0.25 *vs* 252.43 ± 0.47 mOsm/kg for KSOMaa medium (n = 3) in embryo culture dishes (Fig. 3 and Supplemental Table S3). Although the osmolarity of prewarmed embryo culture medium (KSOMaa) in manual group is slightly higher than that in automated group, no significant statistical differences were found in prewarmed IVF or embryo culture media in both groups (Fig. 3 and Supplemental Table S3; *P* > 0.05). This result indicated that the automated preparation did not give rise to severe osmolality fluctuations.

### Effects of automated and manual preparation on IVF and embryo development

**Figure 4:**
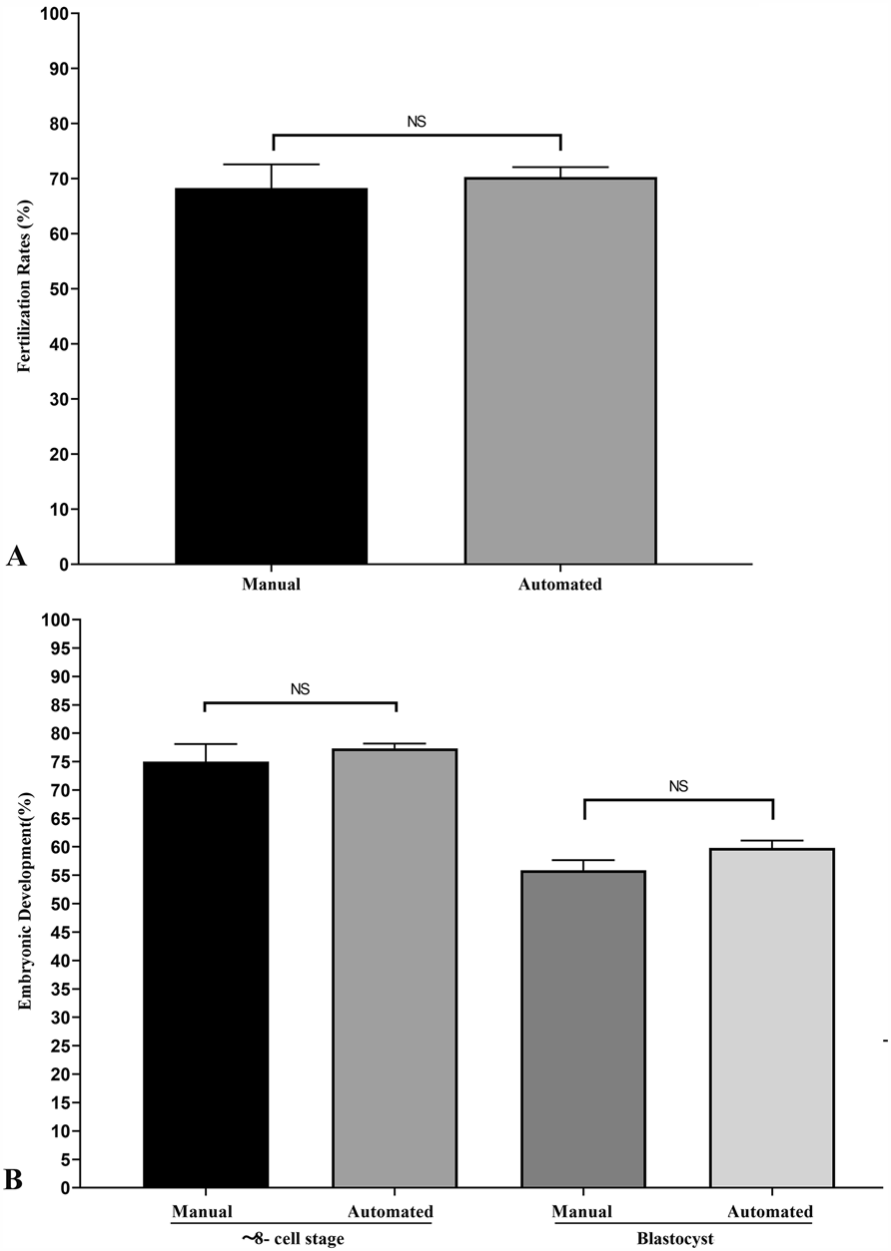
Fertilization rates of mouse oocytes and subsequent embryonic development in manually and automatically prepared IVF dish. A total 123 and 138 of MII oocytes were inseminated in manually and automatically prepared IVF dishes; the fertilization rates were 68.28 ± 4.32% and 70.27 ± 1.83%, respectively. A total 84 and 97 of zygotes (with 2PN) from two types of IVF dishes were correspondingly cultivated in manually and automatically prepared embryo culture dishes; the rates of 8-cell-embryo were 74.99 ± 3.10% and 77.34 ± 0.83%; and the percentages of blastocyst were 55.89 ± 1.78% and 59.83 ± 1.29% between two groups. NS means no significant difference.

A total 123 and 138 of MII oocytes (n = 3) were collected and inseminated in manually and automatically prepared IVF dishes, respectively. There was no statistical difference in their fertilization rates (68.28 ± 4.32% *vs* 70.27 ± 1.83%; Fig. 4A and Supplemental Table S4).

A total 84 and 97 of zygotes with 2PN (n = 3) from two types of IVF dishes were correspondingly cultivated in manually and automatically prepared embryo culture dishes, respectively. There was no significant difference in the rates of 8-cell-embryo (74.99 ± 3.10% *vs* 77.34 ± 0.83%) and blastocyst (55.89 ± 1.78% *vs* 59.83 ± 1.29%; *P* > 0.05) between two groups (Fig. 4B and Supplemental Table S5).

## Discussion

During the IVF process, the techniques mastered by embryologists mainly include the maintenance of a laboratory and equipment, preparation and quality control of culture media and labware, operation of gametes and embryos, and so on^16^. While the impact of variability in embryologists' experience and procedural timings was not directly evident in the embryological and clinical data, but it is clear that the fast-paced nature of these professionals significantly affects the overall efficiency and productivity of IVF laboratory^17^. The preparation of labware is an essential operation in the laboratory and should not be overlooked. This includes the manual preparation of dishes, which is a routine operation that relies on the expertise and experience of the embryologist. The culture dishes prepared by embryologists with different experiences may have differences in layout, size and regularity of droplet. In daily work, a few flaws may occasionally occur in manually prepared culture dishes especially by junior embryologist. However, the senior embryologists could prepare dishes faster than junior embryologists, and the droplet layout in dishes prepared by them may be more regular and droplet volume more uniform. In manually prepared dishes, we sometimes found marked differences in microdroplet sizes, irregular arrangement, occasional merging of adjacent droplets, and some droplets easily spreading to the edge of dishes. However, the sizes of and distances between microdroplets were always consistent in automatically prepared dishes. Furthermore, compared to the automated preparation method, manually controlling the dispensing of medium in IVF and embryo culture dishes can sometimes lead to inconsistent heights or layers of the medium. The qualified rate of manually prepared culture dishes is essentially determined by the work experience of the embryologists, which is influenced by personnel factors. Therefore, our results in Fig. 2 and Supplemental Table S2 indicate that the automated preparation of culture dishes was overall superior to the manual preparation in terms of qualified rate and consistency, including the size, regularity, layout of medium droplets, and height of liquid (oil).

In addition, it is important to highlight that automated preparation system works in a one-touch manner and can be easily mastered by the majority of embryologists. Therefore, automated culture dish preparation may be a more standard alternative to manual operation.

Some procedures in IVF laboratory can result in the changes of osmolality, which can affect embryo development, especially in microdroplet or small-volume approaches. Therefore, embryologists must be aware of the influence of airflow, working-surface temperature and the method of drop preparation^9^, and adjust the protocols according to more reasonable culture methods. Preventing osmolality changes in human clinical practice can reduce stress levels for embryos and improve clinical outcomes^18,19^. Thus, during the preparation of culture dishes, it is important to minimize evaporation to avoid changes in medium osmolality. To test the influence of automated preparation on the osmolality of culture media, we measured the osmolalities of HTF or KSOMaa in culture dishes prepared using automated or manual approaches. We found that the automated preparation did not significantly affect the immediate osmolalities of different media, even the overnight prewarmed media (Fig. 3 and Supplemental Table S3).

In order to shorten the time of in vitro operation and decrease the risks of evaporation, many large IVF centers generally arranges two embryologists to prepare culture dishes; one dispenses medium droplets while another covers the droplets with a certain volume of culture oil by pipetting in time, otherwise the preparation time could be extended too much and result in irreversible increasement of medium osmolality. In our automated preparation system, the two independent manual operations are combined into a single one-step operation. This reduces the exposure time of media in air and prevents water evaporation from medium droplets, ensuring the stability of culture media osmolality (Fig. 3).

In this automated preparation system, several procedures can be preprogrammed for a variety of culture dishes according to different requirements such as IVF and embryo culture. Volume and location of droplets can be precisely controlled through PID algorithm; and the uniform volume and distance between droplets can avoid the mixing of adjacent droplets occasionally caused by manual operation (Fig. 2 and Supplemental Movie S2). The mechanical design of the entire automation equipment derived from the manual operation process, and fully guaranteed the safety and operability of automated manipulation. In addition, preparation of various dishes in batches could substantially shorten the time of in vitro operation (Fig. 1 and Supplemental Table S1), and avoid the serious shift of osmolality (Fig. 3).

Interestingly, the standard deviations (SDs) of the time required for manual preparations (Fig. 1) are much larger than those of automated preparations, including both IVF (2.39 *vs* 1.02 s) and embryo culture dishes (1.41 *vs* 0.40 s). This observation suggests that manual preparation introduces a higher degree of variability in the time taken, primarily due to the influence of personnel factors. Furthermore, while automated preparation of IVF culture dishes is quicker than manual preparation of embryo culture dishes (12.05 ± 1.02 s *vs* 13.06 ± 1.41 s), it was found that the overall time required for preparing IVF dishes was correspondingly longer than that for embryo culture dishes during both manual and automated preparation. This discrepancy in time can primarily be attributed to the volume of the liquid and oil used in the process. It is observed that the larger the volume of medium and oil, the longer the time required for preparation.

Furthermore, to assess the effectiveness of automated preparation system in IVF and embryo cultivation, we compared the rates of fertilization, 8-cell cleavage embryos and blastocysts between the manual and automated procedures. Our results (Fig. 4A) showed that the fertilization rates of automated preparation were equal to that of manual preparation, and the embryos derived from automated preparation system had similar rates of ~ 8- cell cleavage and percentages of blastocysts compared to the manual preparation (Fig. 4). Thus, the automated system obtained almost identical IVF rates and high-quality embryos compared to conventional manual preparations, indicating that it was suitable for IVF and embryo cultivation as an alternative to traditional manual preparation.

What makes this automated preparation system fascinating is its application in different IVF laboratories for preparing various types of dishes. This adaptability could be achieved by changing the end-effector vacuum manipulator, adjusting the size and layout of the motion dish platform, and programming by the pocket programmer. As a result, the automated system can be customized to suit specific needs, ensuring compatibility with different types and sizes of culture dishes used in various IVF clinics, regardless of its scale.

In summary, as a technique easy to master, the automated dish preparation system is suitable for preparing multi-types of culture dishes and is able to reduce the workloads of embryologists. Moreover, the automated dish preparation in batches saves much time of in vitro operation while it is helpful for maintaining the stability of medium osmolality, which is beneficial for embryonic development. This automated system has the potential to provide a standard and repeatable process that partially replaces manual operations, resulting in more stable culture outcomes. It is worth considering the application of this system in human IVF or animal reproductive biology laboratories.

## Materials and methods

### Animals

ICR (Institute of Cancer Research, USA) mice were obtained at 5 to 6 weeks of age from Guangdong Laboratory Animals Center (Guangzhou City, China). All animals were maintained in a 12-h light and 12-h dark cycle in accordance with the Animal Care Guidelines for Use of Animals of Laboratory Animal Center, Guangdong Province Second General Hospital, Guangzhou, China. All experimental protocols were performed after obtaining the approval from the ethical committee of Guangdong Second Provincial General Hospital for animal experiments (No. 2014-KYLLM-065) and all the used methods are reported in accordance with ARRIVE guidelines. Euthanasia was performed by CO2 asphyxiation followed by cervical dislocation according to AVMA guidelines.

### Reagents and materials

Pregnant mare serum gonadotropin (PMSG) and human chorionic gonadotropin (hCG) were purchased from Ningbo Sansheng Hormone Factory (Ningbo City, China). All other chemicals were obtained from Sigma-Aldrich Chemical Co. (St. Louis, MO, USA) unless otherwise stated. All of the consumables including Falcon 3037 organ-culture dish (Corning Inc., Tewksbury, MA, USA), 3.5 cm Nunc polystyrene easydish (Thermo Fisher Scientific Inc., Waltham, MA, USA), medical silcone tube (Siweier Plastic Tech Co., Suzhou, China), medical three-way valves (Beter Medical Equipment Co., Shanghai, China), syringes (WEGO Holding Co., Weihai, China), and pipettes (Thermo Fisher Scientific Inc), are disposable sterile items.

### Working experience of embryologists

All the personnel who participated in this program were senior embryologists with more than 8 years of clinical or animal embryology experience. They were engaged in the dish preparation and embryo cultivation during the whole process.

### Working mechanism of automated culture dish preparation system

**Figure 5:**
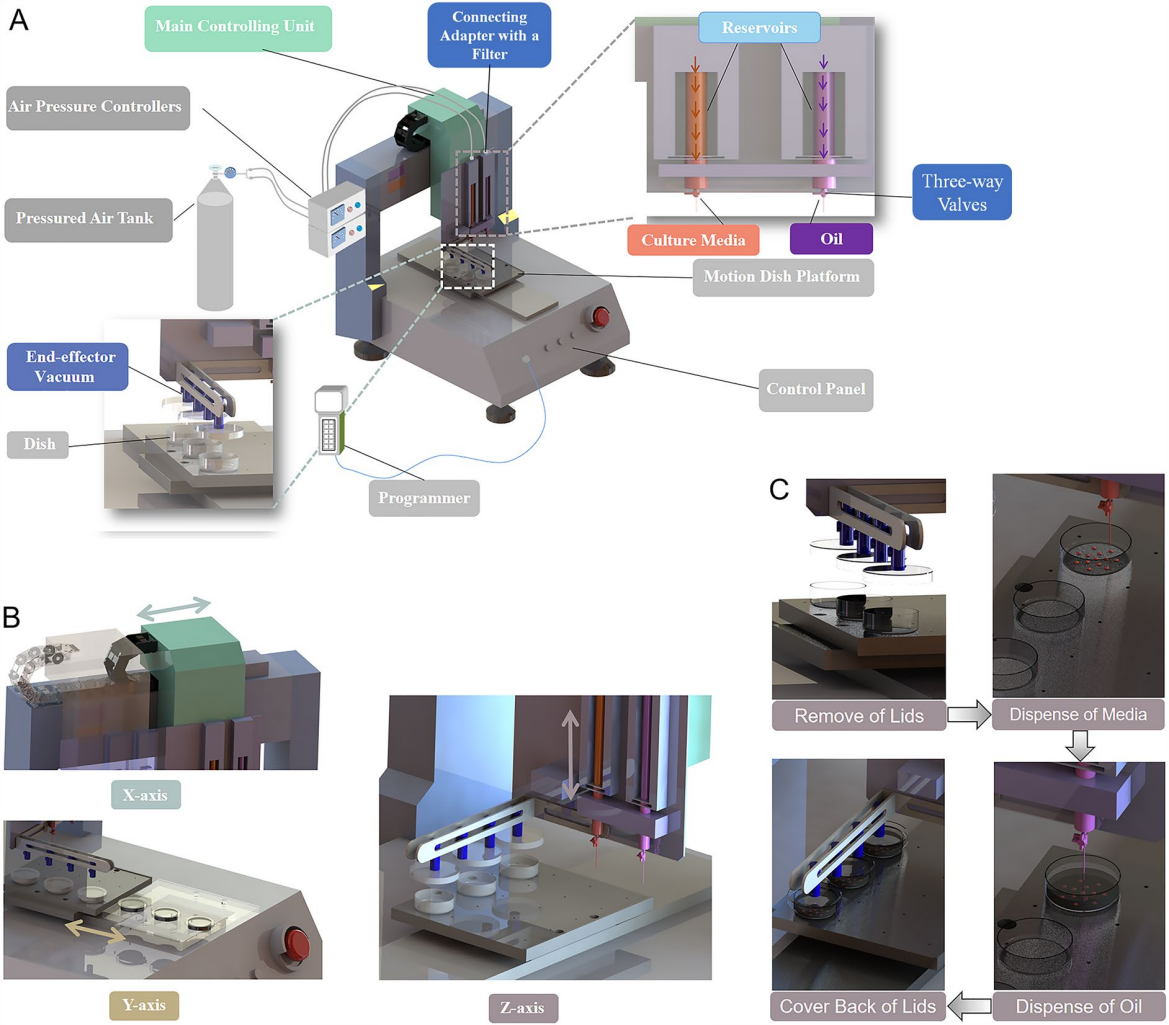
Structural diagram of automated culture dish preparation system. (**A**) Automated culture dish preparation system mainly contains a main controlling unit, two air pressure controllers, a programmer, an end-effector vacuum manipulator and a motion dish platform. (**B**) Motion patterns of the main controlling unit and culture dishes in x-, y- and z- axial directions. (**C**) Full process of automated dish preparation includes the remove of lids, dispense of culture media and oil, and the cover back of lids.

The automated dish preparation system (Fig. 5A) consists of a main controlling unit, two air pressure controllers, a motion dish platform (Jiaheng Intelligent Equipment Co. Shenzhen, China) and an end-effector vacuum manipulator (Taozhuo Automation Equipment Co., Xuzhou, China). The main controlling unit is responsible for controlling the motion space and the pre-setup given positions by initial coordinates and time intervals of different actions in x- and z- axial directions (Fig. 5B); the air pressure controllers accurately control the volume of medium droplet and oil layer; the end-effector vacuum manipulator controls the remove and cover of dish lids (Fig. 5C); the platform is applied to fix the culture dishes by fixture tools and control their movement in y- axial direction (Fig. 5B). The motion space is controlled by a two-axial main controlling unit and a motion platform, which completes the motion of any coordinate in the three-dimensional space (Fig. 5B). The accuracy of the position controlling was achieved through the proportional, integral and derivative (PID) control algorithm (Supplemental Material S1).

The air pressure controllers execute dispensing control through output of air pressure (kPa), time per pulse (second) and inner diameter of dispensing tips (G) proportional to the volume of microdroplet and oil, and accurately control the volumes of medium droplet and oil layer. All controlling parameters of automatically dispensing HTF, KSOMaa and culture oil are listed in Table 1. The source of air pressure comes from clean medical-grade compressed nitrogen gas, with a purity of 99.99% (Praxair Huizhou Industrial Gas Co., Huizhou, China).

**Table 1:** Parameters for controlling the dispensing volumes of IVF and embryo culture media.

	Output of air pressure (kPa)	Type of dispensing tip (G)	Spray type of dispensing tip	Time per pulse (s)	Output volume of droplet (μl)
HTF for IVF	2	23	Circular spray (radius = 2 cm)	2	1000
KSOMaa for embryo culture	2	23	Dot spray	0.5	15
Oil for IVF dish	60	18	Dot spray	2	2000
Oil for embryo culture dish	60	18	Dot spray	2.5	2500

All the parameters including output of air pressure(kPa), spray type of dispensing tip, time per pulse(second) and type of dispensing tip (gauge) for automatically preparing IVF and embryo culture dishes were listed in this table.

### Workflow of automated culture dish preparation system

At first, the power switch was turned on. According to the required volume or droplet size of culture media and oil, and their locations in a variety of culture dishes, the given procedures were preprogrammed by using the Programmer (Fig. 5A), and the procedures were named as IVF Dish and Embryo Culture Dish. The medium reservoir (on the left) and oil reservoir (on the right) were constructed using a syringe and a medical three-way valve. The top openings of these reservoirs were connected to the outlets of air pressure controllers via an adapter (Fig. 5A). Pre-determined volumes of culture media and oil were filled into the medium reservoir and oil reservoir respectively, using the three-way valves, based on the total volumes needed for the preparations (Fig. 5A). And then, a required procedure such as IVF Dish/Embryo Culture Dish was selected on the screen of Programmer. In order to ensure that the pre-setting location of each microdroplet was accurately consistent with the dispensing position, the initial position of dispensing tip was calibrated by programmer before starting the automated operation. Three of IVF or four of embryo culture dishes were then placed onto the fixed positions of platform each time.

Once automated operation started, the end-effector vacuum manipulator automatically moved onto and sucked the lids of culture dishes to upright.

Afterwards, the lids automatically moved to another position and the dispensing process of culture medium automatically started to work. The medium dispensing tip dispensed culture medium to the corresponding positions droplet by droplet in culture dishes according to pre-setting location and volume. Then, the oil dispensing tip immediately moved to the top of dish, the appropriate volume of oil was dispensed to cover the medium droplets completely. Subsequently, the lids automatically returned to original position, the vacuum manipulator automatically turned off and the lids were released to cover the original culture dishes. Once whole procedure was completed, the power switch was finally turned off.

### Preparation of IVF dishes

#### Manual preparation of IVF dishes (HTF medium)

On the work-surface of IVF workstation (airflow was off) (K-system, Denmark) at ambient temperature (22–24 °C), 1.0 ml of HTF medium (Sigma-Aldrich Chemical Co., USA) were pipetted into the central well of Falcon 3037 organ-culture dish (Corning Inc., Tewksbury, MA, USA) by using the eppendorf electronic pipette controller (Eppendorf AG, Germany) with 10 ml serological pipets (Thermo Fisher Scientific Inc., USA) and 2.0 ml oil (Sigma-Aldrich Chemical Co.) was covered over the HTF medium by using an electronic pipette controller with 20 ml serological pipets (ThermoFisher Scientific, USA). The total time of preparing dishes was recorded and calculated by using a timer. The starting time-point was recorded when the lids were removed from culture dishes; the terminal time-point was recorded once the lids were covered back the original culture dishes.

#### Automated preparation of IVF dishes (HTF medium)

Under the same conditions as the manual preparation, according to the required total volume, HTF medium and oil were prefilled into medium reservoir (left) and oil reservoir (right), respectively (Fig. 5A). Once IVF dish preparation started according to the pre-setting procedure, 1.0 ml of HTF medium were automatically dispensed into each central-well of Falcon 3037 polystyrene dishes and 2.0 ml oil was covered over HTF medium (Supplemental Movie S1). The total time was recorded by using a timer. After the whole process was completed, all dishes were transferred into CO_2_ incubator at 37 °C with 5% CO for use.

### Preparation of embryo culture dishes

#### Manual preparation of microdroplet culture dishes (KSOMaa)

On the work-surface of airhood (airflow was off) and under the condition of 22–24 °C, dependent of the number of embryos, thirteen or less of 15 μl KSOMaa (Millipore Co., USA) microdroplets (three of them for washing, the others for embryo culture) were pipetted onto the bottom of 3.5 cm Nunc polystyrene easydish (Thermo Fisher Scientific Inc., Waltham, MA, USA) by using an eppendorf pipette (Research^®^ plus, Germany) with non-sterile 200 μl of tips (Axygen Inc, USA) and 2.5 ml oil was finally covered over the medium droplets by using the electronic pipette controller with 20 ml serological pipets. The total time was recorded by using a timer.

#### Automated preparation of microdroplet culture dishes (KSOMaa)

Under the same conditions as the manual preparation, according to the required volumes, KSOMaa media and oil were prefilled into medium reservoir (left) and oil reservoir (right), respectively. After Embryo Culture Dish process was started according to the pre-setting procedure, 13 of 15 μl KSOMaa microdroplets (Millipore Co., USA) were automatically dispensed onto the bottom of 3.5 cm polystyrene dishes and 2.5 ml oil was finally covered over the medium (Supplemental Movie S2). The required total time was recorded by using a timer. Once the entire preparation process was completed, all dishes were transferred into the 37 °C and 5% CO_2_ incubator for use.

### Compassion of droplet regularities and qualified rates of IVF or culture dishes

After preparing IVF or culture dishes using both manual and automated methods, there may be several situations that indicate the dishes are not up to standard. These situations include abnormal medium and oil layer height in IVF dishes, inconsistent microdroplet size, irregular arrangement of microdroplets, merging of adjacent droplets, and droplets flattening to the dish edge in culture dishes. Conversely, dishes that do not show any of these abnormalities are considered to be qualified. The qualified rate of IVF or culture dishes can be calculated by dividing the number of qualified dishes by the total number of prepared dishes.

### Osmolality measurement of IVF and embryo culture media

IVF and embryo culture dishes prepared by automated or manual methods were prewarmed overnight in CO_2_ incubator at 37 °C with humidified 5% CO_2_ in air and the osmolalities of HTF and KSOMaa media collected from IVF and culture dishes before putting into or after pre-warming 18 h (overnight) in CO_2_ incubator were measured by an osmometer (OSMOMAT030 Cryoscopic Osmometer, Germany). At least 50 µl of sample was collected from the central well of IVF dish or at least 4 microdroplets from the embryo culture dish for measuring their osmolality, and each measurement required three repetitions.

IVF dishes containing HTF were prepared by manual and automated procedures, resulting in four testing groups: the osmolalities of HTF in manually and automatically prepared IVF dish were immediately measured (0 h); the osmolalities of HTF in manually and automatically prepared IVF dish were measured overnight (18 h);

Embryo culture dishes containing KSOMaa were prepared by manual and automated procedures, resulting in four testing groups: the osmolalities of KSOMaa in manually and automatically prepared embryo culture dish were measured immediately (0 h) and the osmolalities of KSOMaa in manually and automatically prepared embryo culture dish were measured overnight (18 h).

### In vitro fertilization (IVF) and embryo culture

Epididymal spermatozoa were collected from the cauda epididymis of 8- to 10-week-old ICR mice and incubated in HTF medium for 30 min at 37 °C in air containing 5% CO_2_.

The IVF method was similar to that described by Chaigne^20^ with a minor modification. In detail, the females injected with PMSG and hCG were euthanized and the contents of their oviducts transferred into a drop of HTF. The ampullae were tore and egg clutches were released into a manually or automatically prepared IVF dish and then about 10 μl of the sperm were added. The oocytes and sperm were co-incubated in two types of IVF dishes for 4–6 h at 37 °C in 5% CO_2_ in air; the eggs were then washed to remove the excess of sperm. The fertilization in two types of IVF dishes was evaluated and compared according to the number of each inseminated oocyte. Oocytes with two pronuclei and a clear second polar body were considered normally fertilized.

All zygotes from two types of IVF dishes were correspondingly transferred into a manually or automatically prepared embryo culture dish and cultivated at 37 °C in 5% CO_2_ in air for 3.5 days. The percentage of cleaved oocytes was assessed at 24 h after insemination. From day 1 to day 3.5 following insemination, the embryos were observed under a microscope to compare the percentages of embryonic development to 8-cell stage and blastocyst in each group respectively.

### Statistical analysis

Data were analyzed using Prism 6.0 (GraphPad Software Inc., La Jolla, CA, USA). Preparation time per dish, qualified rates of culture dishes, osmolalities of media, rates of fertilization, 8-cell cleavage embryo and blastocyst were expressed as mean ± standard deviation (SD) and compared using one-way analysis of variance (ANOVA). A p value of < 0.05 was considered statistically significant.

### Supplementary Information

Supplementary Information.

Supplementary Video S1.

Supplementary Video S2.

Supplementary Table S1.

Supplementary Table S2.

Supplementary Table S3.

Supplementary Table S4.

Supplementary Table S5.

## Data Availability

All data generated or analysed during this study are included in this published article (and its Supplementary Information files).
