# Supplementary material for: Evaluation of an automated dish preparation system for IVF and embryo culture using a mouse mode
Source: Sci Rep. 2023 Oct 1;13:16490. doi: 10.1038/s41598-023-43665-y (PMC10543539; doi:10.1038/s41598-023-43665-y)
Supplement: Supplementary file 1 — Supplementary Information. [file 41598_2023_43665_MOESM1_ESM.docx]

**Mechanical Controlling Mechanism of the Automated Preparation**

PID stands for Proportional-Integral-Derivative, which is a control algorithm commonly used in automation and control systems. It is commonly used in the context of solution dispense. During solution dispense, PID control is used to regulate the flow rate or volume of the solution being dispensed. The PID controller could continuously monitor the error, which is the difference between the desired setpoint and the actual dispense value^1,2^.

The control method of the operation system during the automated preparation of medium and culture oil executes the traditional PID control according to the following equation,

$$u(t)=K_{P}[e(t)+\frac{1}{T_{I}}\int_{0}^{t} e(t)dt+T_{D}\frac{de(t)}{dt}]+u_{0}$$

Where $e(t)$ is the deviation between set-point and reference signal point variable, $K_{P}$is the scale factor, $T_{I}$ is the integration time constant, $T_{D}$ is the differential time constant, $t$ is the time interval from the start of adjustment to the output of the current control variable and $u_{0}$ is the input control signal of the actuator is a fixed value during the control process.

Reference:

1 Strm, K. J. & Hgglund, T. PID controllers: Theory, Design and Tuning. *instrument society of america research triangle park nc* (1995).

2 Jiandong, C., Endian, H. U., Wenxian, Z. & Dong, Z. Design and implementation of liquid-level control system based on PID parameters self-tuning. *Modern Electronics Technique* (2016).
