# Supplementary material for: Evaluation of an automated dish preparation system for IVF and embryo culture using a mouse mode
Source: Sci Rep. 2023 Oct 1;13:16490. doi: 10.1038/s41598-023-43665-y (PMC10543539; doi:10.1038/s41598-023-43665-y)
Supplement: Supplementary file 4 — Supplementary Table S1. [file 41598_2023_43665_MOESM4_ESM.docx]

| Type of Dish | Replicates of Testing | Manual Preparation | | | Automated Preparation | | |
| --- | --- | --- | --- | --- | --- | --- | --- |
|  |  | No. of Dish | Total Time(second) | Average Time(second) | No. of Dish | Total Time(second) | Average Time(second) |
| IVF Dish | 1 | 4 | 75 | 18.75 | 5 | 53 | 10.60 |
|  | 2 | 5 | 81 | 16.20 | 5 | 62 | 12.40 |
|  | 3 | 5 | 80 | 16.00 | 6 | 68 | 11.33 |
|  | 4 | 4 | 74 | 18.50 | 4 | 56 | 14.00 |
|  | 5 | 6 | 86 | 14.33 | 7 | 81 | 11.57 |
|  | 6 | 7 | 80 | 11.43 | 5 | 63 | 12.60 |
|  | 7 | 8 | 125 | 15.63 | 6 | 70 | 11.67 |
|  | 8 | 7 | 123 | 17.57 | 5 | 61 | 12.20 |
|  |  | | Mean | 16.05 |  | Mean | 12.05 |
|  |  |  | SD | 2.39 |  | SD | 1.02 |
| Embryo Culture Dish | 1 | 3 | 38 | 12.67 | 4 | 39 | 9.75 |
|  | 2 | 4 | 40 | 10.00 | 3 | 28 | 9.33 |
|  | 3 | 5 | 68 | 13.60 | 3 | 26 | 8.67 |
|  | 4 | 3 | 40 | 13.33 | 4 | 39 | 9.75 |
|  | 5 | 5 | 66 | 13.20 | 4 | 38 | 9.50 |
|  | 6 | 5 | 64 | 12.80 | 3 | 28 | 9.33 |
|  | 7 | 4 | 57 | 14.25 | 5 | 44 | 8.80 |
|  | 8 | 3 | 44 | 14.67 | 4 | 37 | 9.25 |
|  |  | | Mean | 13.06 |  | Mean | 9.30 |
|  |  |  | SD | 1.41 |  | SD | 0.40 |

**Supplemental Table S1.** Comparison of the Required Time for Dish Preparation
