# Supplementary material for: Evaluation of an automated dish preparation system for IVF and embryo culture using a mouse mode
Source: Sci Rep. 2023 Oct 1;13:16490. doi: 10.1038/s41598-023-43665-y (PMC10543539; doi:10.1038/s41598-023-43665-y)
Supplement: Supplementary file 5 — Supplementary Table S2. [file 41598_2023_43665_MOESM5_ESM.docx]

| Type of Dish | Replicates | Manual Preparation | | | Automated Preparation | | |
| --- | --- | --- | --- | --- | --- | --- | --- |
|  |  | No. of Dish | No. of Qualified Dish | Qualified Rates (%) | No. of Dish | No. of Qualified Dish | Qualified Rates (%) |
| IVF Dish | 1 | 15 | 14 | 93.33% | 18 | 18 | 100.00% |
|  | 2 | 28 | 27 | 96.43% | 24 | 24 | 100.00% |
|  | 3 | 33 | 31 | 93.94% | 27 | 26 | 96.30% |
|  |  |  | Mean | 94.57% |  | Mean | 98.77% |
|  |  |  | SD | 1.64% |  | SD | 2.14% |
| Embryo Culture Dish | 1 | 23 | 15 | 65.22% | 36 | 36 | 100.00% |
|  | 2 | 16 | 10 | 62.50% | 24 | 24 | 100.00% |
|  | 3 | 25 | 16 | 64.00% | 20 | 20 | 100.00% |
|  |  |  | Mean | 63.91% |  | Mean | 100.00% |
|  |  |  | SD | 1.36% |  | SD | 0.00% |

**Supplemental Table S2.** Qualified Rates of Dishes by Manual and Automated Preparations
