# Supplementary material for: Evaluation of an automated dish preparation system for IVF and embryo culture using a mouse mode
Source: Sci Rep. 2023 Oct 1;13:16490. doi: 10.1038/s41598-023-43665-y (PMC10543539; doi:10.1038/s41598-023-43665-y)
Supplement: Supplementary file 6 — Supplementary Table S3. [file 41598_2023_43665_MOESM6_ESM.docx]

**Supplemental Table S3.** Osmolality Changes in IVF and Embryo Culture Dishes between Manual and Automated Preparation

| Group | Replicate of Testing | HTF (mOsm/Kg) | | KSOMaa (mOsm/Kg) | |
| --- | --- | --- | --- | --- | --- |
|  |  | 0 hour | Overnight | 0 hour | Overnight |
| Manual | 1 | 281.8 | 283.5 | 251.8 | 253.2 |
|  | 2 | 280.5 | 282.1 | 250.2 | 252.9 |
|  | 3 | 281.6 | 283.3 | 251.1 | 253.4 |
|  | Mean | 281.3 | 282.97 | 251.03 | 253.17 |
|  | SD | 0.7 | 0.76 | 0.8 | 0.25 |
| Automated | 1 | 281.1 | 282.9 | 250.9 | 252.6 |
|  | 2 | 281.6 | 283.2 | 251.5 | 251.9 |
|  | 3 | 280.7 | 283.4 | 251.7 | 252.8 |
|  | Mean | 281.13 | 283.17 | 251.37 | 252.43 |
|  | SD | 0.45 | 0.25 | 0.42 | 0.47 |
