# Supplementary material for: Evaluation of an automated dish preparation system for IVF and embryo culture using a mouse mode
Source: Sci Rep. 2023 Oct 1;13:16490. doi: 10.1038/s41598-023-43665-y (PMC10543539; doi:10.1038/s41598-023-43665-y)
Supplement: Supplementary file 7 — Supplementary Table S4. [file 41598_2023_43665_MOESM7_ESM.doc]

**Supplemental Table S4. Fertilization Rates of Mouse Oocytes in Manually and Automatically Prepared IVF Dishes**

| Group | Replicates | No. of Oocytes | No. of Zygotes | Fertilization Rates (%) | Total Fertilization Rates (%) |
| --- | --- | --- | --- | --- | --- |
| Manual  Automated | 1  2  3  1  2  3 | 40  42  41  46  45  47 | 26  28  30  32  31  34 | 65.00  66.67  73.17  69.57  68.89  72.34 | 68.28 ± 4.32^a^  70.27 ± 1.83^a^ |

Percentages are based on the total number of oocytes. Values are mean ± SD (three replicates) unless otherwise stated and values in the same column with same superscripts (a) means no statistical different (*P* >0.05).
