# Supplementary material for: Evaluation of an automated dish preparation system for IVF and embryo culture using a mouse mode
Source: Sci Rep. 2023 Oct 1;13:16490. doi: 10.1038/s41598-023-43665-y (PMC10543539; doi:10.1038/s41598-023-43665-y)
Supplement: Supplementary file 8 — Supplementary Table S5. [file 41598_2023_43665_MOESM8_ESM.doc]

**Supplemental Table S5.** **Subsequent Embryonic Development in Manually and Automatically Prepared Culture Dishes**

| Group | Replicates | No. of zygotes | Embryos Developed to～8- cell stage | | | Embryos Developed to Blastocyst | | |
| --- | --- | --- | --- | --- | --- | --- | --- | --- |
|  |  |  | No. | Percentage (%) | Total Percentage (%) | No. | Percentage (%) | Total Percentage (%) |
| Manual  Automated | 1  2  3  1  2  3 | 26  28  30  32  31  34 | 19  22  22  25  24  26 | 73.08%  78.57%  73.33%  78.13%  77.42%  76.47% | 74.99 ± 3.10^a^  77.34 ± 0.83^a^ | 14  16  17  19  19  20 | 53.85  57.14  56.67  59.38  61.29  58.82 | 55.89 ± 1.78^b^  59.83 ± 1.29^b^ |

Percentages are based on the total number of zygotes. Values are mean ± SD (three replicates) unless otherwise stated and values in the same column with same superscripts (a or b) means no statistical different (*P* >0.05).
